# 

**Published:** 1888-06

**Authors:** 


					﻿Entered at the Post Office, at Austin, Texas, as Second Class Mail Matter
Vol. III
JUNE, 1888.
NO. 12
DANIEL'S
MEDICAL
JOURNAL
Steam Book and Jor Printer, Austin, Texas
				

